# Mindfulness Intervention for Health Information Avoidance in Older Adults: Mixed Methods Study

**DOI:** 10.2196/69554

**Published:** 2025-01-28

**Authors:** Chenyu Gu, Liquan Qian, Xiaojie Zhuo

**Affiliations:** 1 School of Journalism and Communication Minjiang University Fuzhou China; 2 School of Arts and Media Wuhan College Wuhan China

**Keywords:** health information avoidance, cyberchondria, self-determination theory, mindfulness, elderly

## Abstract

**Background:**

The global aging population and rapid development of digital technology have made health management among older adults an urgent public health issue. The complexity of online health information often leads to psychological challenges, such as cyberchondria, exacerbating health information avoidance behaviors. These behaviors hinder effective health management; yet, little research examines their mechanisms or intervention strategies.

**Objective:**

This study investigates the mechanisms influencing health information avoidance among older adults, emphasizing the mediating role of cyberchondria. In addition, it evaluates the effectiveness of mindfulness meditation as an intervention strategy to mitigate these behaviors.

**Methods:**

A mixed methods approach was used, combining quantitative and qualitative methodologies. Substudy 1 developed a theoretical model based on self-determination theory to explore internal (positive metacognition and health self-efficacy) and external (subjective norms and health information similarity) factors influencing health information avoidance, with cyberchondria as a mediator. A cross-sectional survey (N=236) was conducted to test the proposed model. Substudy 2 involved a 4-week mindfulness meditation intervention (N=94) to assess its impact on reducing health information avoidance behaviors.

**Results:**

Study 1 showed that positive metacognition (β=.26, *P*=.002), health self-efficacy (β=.25, *P*<.001), and health information similarity (β=.29, *P*<.001) significantly predicted health information avoidance among older adults. Cyberchondria mediated these effects: positive metacognition (effect=0.106, 95% CI 0.035-0.189), health self-efficacy (effect=0.103, 95% CI 0.043-0.185), and health information similarity (effect=0.120, 95% CI 0.063-0.191). Subjective norms did not significantly predict health information avoidance (β=‒.11, *P*=.13), and cyberchondria did not mediate this relationship (effect=‒0.045, 95% CI ‒0.102 to 0.016). Study 2 found that after the 4-week mindfulness intervention, the intervention group (group 1: n=46) exhibited significantly higher mindfulness levels than the control group (group 2: n=48; M_group1_=4.122, M_group2_=3.606, *P*<.001) and higher levels compared with preintervention (M_t2_=4.122, M_t1_=3.502, *P*<.001, where t1=preintervention and t2=postintervention). However, cyberchondria levels did not change significantly (M_t1_=2.848, M_t2_=2.685, *P*=.18). Nevertheless, the results revealed a significant interaction effect between mindfulness and cyberchondria on health information avoidance (effect=‒0.357, *P*=.002, 95% CI ‒0.580 to ‒0.131), suggesting that mindfulness intervention effectively inhibited the transformation of cyberchondria into health information avoidance behavior.

**Conclusions:**

This study reveals the role of cyberchondria in health information avoidance and validates mindfulness meditation as an effective intervention for mitigating such behaviors. Findings offer practical recommendations for improving digital health information delivery and health management strategies for older adults.

## Introduction

### Background

With the global aging population and the rise of digital health information, the health information behavior of older adults has become a critical focus [1]. The adoption of internet and age-friendly technologies has enabled older adults to seek health information online. However, the overwhelming volume and inconsistent quality of information, combined with lower information literacy among older adults, often hinder efficient access to reliable health information, posing challenges to their health management.

Health information avoidance is a growing concern [2]. It involves individuals deliberately avoiding health-related information, often driven by anxiety and uncertainty, which can lead to severe health consequences [3]. Given their greater need for health information, avoidance behaviors in older adults can hinder effective health management and delay timely diagnosis or treatment [4]. However, research on its causes and interventions remains limited. Cyberchondria, characterized by excessive health information searching, is another critical issue. It disrupts daily activities and exacerbates health anxiety, despite being initially aimed at reducing uncertainty [5]. While it has been linked to problems like internet addiction and sleep disorders, its connection to health information avoidance remains underexplored [6,7]. This study posits that cyberchondria amplifies anxiety, which in turn motivates information avoidance as a self-protection mechanism [8]. Viewing cyberchondria as a psychological driver of health information avoidance offers valuable insights into this behavior.

This study introduces mindfulness meditation as a potential intervention. Rooted in Buddhist traditions, mindfulness has been shown to reduce anxiety and improve emotional regulation and cognitive flexibility [9,10]. By applying mindfulness meditation with older adults, this study evaluates its effects on cyberchondria and health information avoidance, providing a cost-effective health management strategy.

In summary, this study aims to explore the psychological mechanisms underlying health information avoidance among older adults, with a particular focus on cyberchondria as a mediating factor. Drawing from self-determination theory, we propose a model that incorporates both internal (positive metacognition and health self-efficacy) and external (subjective norms and health information similarity) factors to explain how they contribute to health information avoidance. Furthermore, we aim to evaluate the effectiveness of mindfulness meditation as a practical intervention to mitigate the impact of cyberchondria on information avoidance behaviors. This study seeks to address the following two research questions:

1. What are the formation pathways of health information avoidance among older adults?
2. Can mindfulness meditation effectively intervene in cyberchondria and reduce health information avoidance behaviors among older adults?

By addressing these questions, this research intends to contribute theoretical and practical insights to enhance older adults’ health management capabilities in the digital era.

### Theory Background and Hypothesis Development

#### Health Information Avoidance Among Older Adults

In the context of global digitalization and an aging population, the health information behavior of older adults has become a critical topic in health management [11]. Health information avoidance, the deliberate decision to avoid health-related information, is particularly prevalent among older adults due to lower information literacy, anxiety about health issues, and fear of disease severity [12]. This behavior poses significant risks, such as missed medical advice or delayed disease prevention and treatment, as older adults often experience poorer health than younger individuals [1].

Although avoidance may temporarily reduce stress, it often exacerbates anxiety by failing to address underlying health concerns and increasing uncertainty [13,14]. It also undermines proactive health management, reducing engagement in health-promoting behaviors and increasing the risk of chronic diseases [15]. Despite growing research, key gaps remain, particularly in understanding how external environments interact with psychological factors and in developing targeted interventions for older adults. Cyberchondria, defined as repeated health searches that heighten anxiety, offers a valuable framework for understanding avoidance behaviors [16]. It highlights the uncertainty and negative emotions triggered by health information, which align with the drivers of avoidance. Incorporating this framework can help explain the anxiety-avoidance cycle in older adults and support the development of targeted interventions to break this cycle.

#### Cyberchondria

Cyberchondria refers to compulsive and excessive searches for health-related information, often intended for self-reassurance. However, these searches usually provide only temporary relief, with anxiety often worsening during and after the process. Despite its negative consequences, such as harm to mental health, overmedicalization, and susceptibility to medical scams, cyberchondria has become a significant public health concern with the rise of mobile internet use [17,18].

Research on cyberchondria highlights 2 main areas. First, contributing factors include low self-esteem, anxiety sensitivity, low tolerance for uncertainty, and high neuroticism [19-21]. Second, its negative impacts include reduced trust in doctors, increased self-treatment behaviors, medical errors, lower quality of life, and wasted health care resources [22,23]. It has also been linked to problematic internet use [24]. Despite these findings, little research has examined the link between cyberchondria and health information avoidance. This study aims to fill this gap by exploring how cyberchondria contributes to avoidance behaviors, providing new insights into health behaviors [25,26]. Understanding this connection is crucial for designing interventions to help vulnerable populations better manage health information and reduce the negative effects of cyberchondria.

#### Self-Determination Theory

Self-determination theory (SDT), proposed by Deci and Ryan [27], explains human motivation and behavior through the fulfillment of 3 psychological needs: competence, relatedness, and autonomy. Behavior is influenced by internal motivation, driven by psychological cognition, and external motivation, shaped by external pressures or rewards [28]. This dual perspective has been widely applied in health psychology, effectively explaining behaviors such as the adoption of personal health record systems driven by autonomous traits (internal factors) and compulsive social media use influenced by social interaction (external factors) [29-31].

Although extensively applied, SDT has not been used to explain cyberchondria. By integrating internal motivations and external environments, SDT provides a valuable framework for understanding its development and guiding effective health interventions [32]. Responding to calls for further exploration of cyberchondria’s antecedents and consequences [33], this study uses SDT to analyze the factors influencing cyberchondria and its connection to health information avoidance.

#### Cyberchondria and Health Information Avoidance

Cyberchondria is characterized by compulsive and frequent internet-based searching for health-related information, which intensifies individuals’ anxiety and concerns about their health. This behavior often leads to cognitive and emotional responses, such as excessive worry about illness and overinterpretation of symptoms, ultimately impacting health behaviors [34]. While cyberchondria has been linked to behaviors like internet addiction, evidence suggests that internet addiction may eventually result in information avoidance. Individuals with cyberchondria often experience heightened anxiety and negative emotional responses when exposed to health-related information [35]. These negative emotions reinforce their fear of health issues, leading to a hypersensitivity toward disease-related information. Based on protection motivation theory, individuals may perceive excessive health information as a source of emotional distress and adopt avoidance behaviors to reduce anxiety and stress [36]. Furthermore, individuals with cyberchondria often interpret ambiguous or uncertain information as a potential threat. This heightened threat perception can result in strong psychological resistance, causing them to proactively reduce exposure to health information to avoid discomfort. Over time, this behavior manifests as health information avoidance [37]. In light of these mechanisms, the following hypotheses are proposed in this study:

H1: Cyberchondria positively influences the health information avoidance.

#### Internal Factors: Positive Metacognition and Health Self-Efficacy

Next, we explore the factors that lead to cyberchondria in older adults. According to the SDT mentioned earlier, an individual’s behavioral intention is influenced by internal cognitive factors. This study selects positive metacognition as one of the key internal factors influencing cyberchondria. Metacognition is commonly defined as “thinking about thinking” and encompasses the beliefs, strategies, and methods individuals use to regulate their internal cognitive processes [38]. Metacognitive beliefs can be categorized as either positive or negative. Positive metacognition refers to the belief that focusing on health issues and extensively thinking about them enhances one’s sense of safety and control. This construct is widely used to explain health anxiety [39].

Research has shown that positive metacognition effectively predicts attentional biases toward health-related information. Individuals with strong positive metacognitive tendencies are more vigilant about their health and more likely to seek health-related information [40]. Such individuals often believe that worrying about their health and gaining more knowledge about illnesses provide a sense of security. This biased thinking pattern drives compulsive health information-seeking behaviors, which are hallmarks of cyberchondria [41]. However, excessive information-seeking often leads to heightened anxiety, cognitive overload, and feelings of inadequacy in managing one’s health, which can result in health information avoidance as a coping mechanism. Therefore, this study hypothesizes that positive metacognition positively influences an individual’s likelihood of developing cyberchondria, which, in turn, leads to health information avoidance. Based on this logic, the following research hypotheses are proposed:

H2a: Positive metacognition positively influences cyberchondria.

H2b: Cyberchondria mediates the effect of positive metacognition on health information avoidance.

The concept of self-efficacy was first introduced by Bandura [42] in 1977, referring to an individual’s confidence and belief in their ability to achieve specific behavioral goals. Ajzen [43] later incorporated it into the theory of planned behavior, which suggests that self-efficacy is a significant predictor of an individual’s behavioral intention. Numerous studies have since confirmed that self-efficacy is an important component of the internal cognitive system, significantly affecting personal behavior and decision-making [44]. Self-efficacy has been widely studied in the field of health behaviors [45], where it is often referred to as health self-efficacy. According to Lee et al [46], health self-efficacy refers to an individual’s confidence in their ability to manage their health. Individuals with higher levels of health self-efficacy are more likely to feel capable of addressing health issues independently and have a stronger inclination toward self-healing. This self-healing tendency reduces communication with health care professionals and encourages individuals to search for health and treatment-related information for self-treatment [47]. Furthermore, the tendency toward self-healing has been shown to be significantly associated with cyberchondria [22]. Therefore, this study speculates that individuals with higher levels of health self-efficacy are more inclined to gather health-related information through internet-based channels, which may, in turn, lead to a tendency toward cyberchondria, causing these users to become trapped in a health information bubble. Based on this reasoning, the following research hypotheses are proposed:

H3a: Health self-efficacy positively influences cyberchondria.

H3b: Cyberchondria mediates the effect of health self-efficacy on health information avoidance.

#### External Factors: Health Information Similarity and Subjective Norms

Health information similarity refers to the consistency or resemblance in the content and structure of health information encountered on digital platforms, often driven by personalized recommendation algorithms tailored to users’ preferences and behaviors [48,49]. These algorithms frequently expose users to overlapping health content, which can create cognitive confusion and negative emotional responses. For instance, similar symptom descriptions across diseases make it difficult to differentiate conditions, increasing uncertainty and anxiety [50]. This anxiety often triggers more frequent searches, leading to information overload and reinforcing a cycle of heightened anxiety.

While previous studies link information similarity to health anxiety, its impact on cyberchondria remains underexplored. Repeated exposure to overlapping health content can cause individuals to misinterpret ordinary symptoms such as severe illnesses, further amplifying uncertainty and intensifying cyberchondria [51]. This exacerbates excessive information searching and, in turn, contributes to health information avoidance. Based on this reasoning, the following hypotheses are proposed:

H4a: Health information similarity positively influences cyberchondria.

H4b: Cyberchondria mediates the effect of health information similarity on health information avoidance.

Subjective norms refer to the perceived social pressure or expectations from others, reflecting the social acceptance of a particular behavior. Both the risk information seeking and processing model and the theory of planned behavior emphasize that social factors (subjective norms) significantly influence an individual’s intention to seek health information [52]. In this study, subjective norms specifically refer to the expectations from important others, such as family and friends, regarding an individual’s health information-seeking behavior. When individuals perceive strong interest or encouragement from their social circle, subjective norms can create pressure to continue searching for health information. This social pressure often leads individuals to feel a heightened sense of responsibility, causing them to persist in health information searches despite experiencing anxiety and discomfort. Such pressure can exacerbate anxiety, trapping individuals in a cycle of frequent searches and information overload [53]. Furthermore, subjective norms can make it difficult for individuals to exercise self-restraint, increasing doubts about their health and ultimately contributing to cyberchondria.

Subjective norms may also worsen cyberchondria by influencing self-perception. When individuals believe that those around them place great importance on health information searching, they may see not engaging in such behavior as irresponsible, further intensifying their psychological burden and health anxiety [54]. To alleviate this emotional distress, individuals may choose to avoid health information altogether, attempting to reduce the psychological burden caused by social pressure. Based on this reasoning, the following hypotheses are proposed:

H5a: Subjective norms positively influence cyberchondria.

H5b: Cyberchondria mediates the effect of subjective norms on health information avoidance.

#### Mindfulness as a Proactive Psychological Intervention Method

The resource model of self-control and ego depletion theory posits that self-control tasks, such as compulsive health information searches, deplete cognitive resources (“ego depletion”), impairing subsequent performance and leading to avoidance of related activities like processing additional health information [55,56]. However, self-control can be improved through consistent training. Mindfulness, rooted in Buddhist traditions, is defined as present-focused attention and nonjudgmental acceptance [57]. It includes 4 elements: being present, attention, awareness, and acceptance [58]. Mindfulness training enhances self-control, reduces cognitive depletion, and improves emotional regulation, making it effective for addressing anxiety, depression, and stress [59,60].

In the context of cyberchondria, individuals experience intense anxiety due to excessive focus on health information, leading to repeated searches that amplify uncertainty and anxiety. This cycle depletes cognitive resources, increasing health information avoidance. Mindfulness training helps break this cycle by enhancing awareness and acceptance, thereby reducing anxiety and distress [61]. Improved emotional regulation allows individuals to manage anxiety without excessive searching, disrupting the “anxiety–information searching–greater anxiety–information avoidance” loop. In addition, mindfulness promotes cognitive flexibility, helping individuals identify and resist cognitive biases common in cyberchondria, such as misinterpreting ordinary symptoms as severe illnesses [62]. By encouraging observation over reaction, mindfulness reduces health anxiety and prevents avoidance behaviors stemming from excessive concern about health information.

H6a: Mindfulness training significantly reduces individuals’ levels of cyberchondria.

H6b: Mindfulness training negatively moderates the positive effect of cyberchondria on health information avoidance.

### Conceptual Model

**Figure 1:**
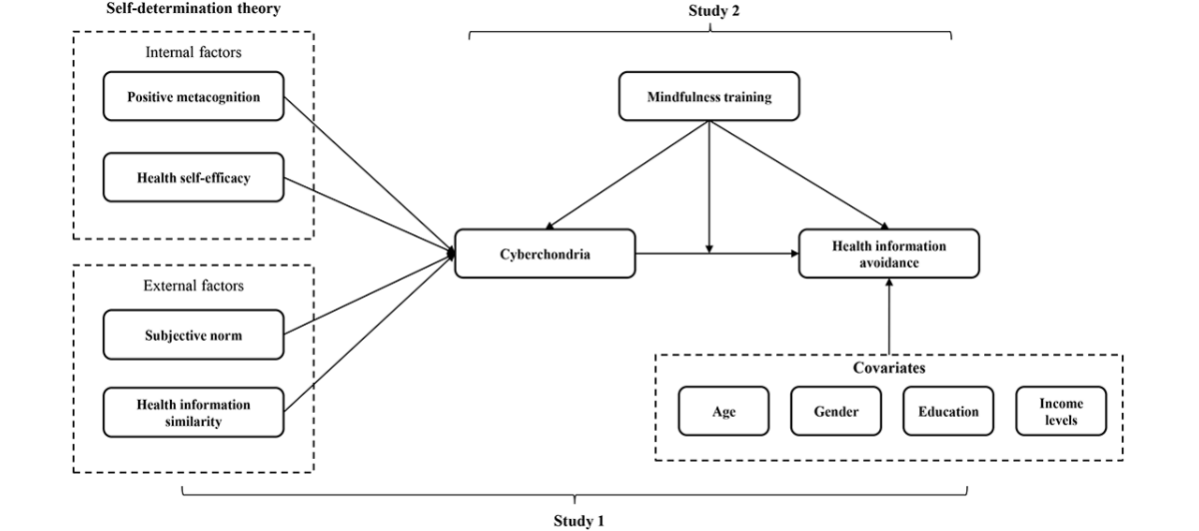
Conceptual model of health information avoidance among older adults.

In summary, this study consists of 2 substudies. Study 1 aims to explore the causes of cyberchondria among older adults in the digital age and the impact model of health information avoidance behavior among older adults. Study 2 aims to validate the intervention effects of mindfulness training on cyberchondria and health information avoidance behavior in older adults. The specific research models are shown in Figure 1.

## Methods

### Study 1

#### Participants and Procedure

Participants for study 1 were primarily recruited through health communities and social media platforms commonly used by older adults, such as WeChat Moments (Tencent), Douyin (the Chinese version of TikTok, ByteDance), and Jinri Toutiao (Today’s Headlines). The target population included individuals aged 60 years and older who were capable of independently completing internet-based questionnaires. To enhance sample diversity and ensure an adequate sample size, additional outreach was conducted through local community networks, such as senior activity centers.

Data for study 1 was collected through internet-based survey platform. Before the survey began, the researchers clearly informed all participants about the purpose of the study and assured them that all responses would be strictly confidential and used solely for academic purposes. The survey was conducted anonymously, with no personal information being disclosed. Participants were also informed of the importance of providing truthful responses and were reminded of their right to withdraw from the survey at any time without any conditions. Informed consent was obtained from all participants.

Initially, 328 questionnaires were collected, with 92 invalid responses excluded, resulting in 236 valid questionnaires (mean age 63.93 years; 91, 38.56% males; 145, 61.44% females). Demographic details are provided in the Multimedia Appendix 1. The response rate of valid questionnaires was 71.95%. The criteria for selecting valid questionnaires were correctly answering screening questions, a total response time of more than 1 minute, and not selecting the same response option for 9 consecutive items.

#### Measurements

Study 1 measured the following latent variables: health information avoidance [63], cyberchondria [64], positive metacognition [65], health self-efficacy [66], subjective norms, and health information similarity [67,68]. The detailed measurement scales can be found in Multimedia Appendix 1. To ensure validity, all measurement tools were adapted from established research and adjusted to fit the context of health information dissemination. The average score of the items in each scale was used to represent each latent variable, and all questionnaires were measured using a 5-point Likert scale. A pretest was also conducted with 10 volunteers to ensure that all items were clearly stated and accurately understood.

#### Measurement of Model

Since this study is exploratory in nature, the research model, although built based on theoretical and logical reasoning, does not originate from an existing model. Therefore, it is appropriate to use “Smart PLS” for partial least squares (PLS) path analysis () to test the research model in this context [69].

Reliability and validity testing. As shown in Table 1, the factor loadings for all measurement items ranged from 0.728 to 0.950, indicating that all items met the retention criteria. In addition, the Cronbach α values for each latent variable ranged from 0.870 to 0.915, and the composite reliability (CR) values for all latent variables were greater than the acceptable threshold of 0.7, indicating that the reliability of the scales was satisfactory. Furthermore, the average variance extracted (AVE) values for all variables exceeded the standard acceptance value of 0.5, demonstrating adequate convergent validity. The variance inflation factor (VIF) values for all factors were below 10, indicating that there were no multicollinearity issues in the scales used in this study [70,71].

**Table 1:** Reliability and convergent validity results of latent variables.

Latent variable and items		Factor loadings	VIF^a^	Cronbach α values		CR^b^		AVE^c^	
**Health information avoidance**					.912		.925		.739
	HIA^d^ 1	.841	2.608						
	HIA 2	.903	3.805						
	HIA 3	.803	2.056						
	HIA 4	.854	2.461						
	HIA 5	.894	2.929						
**Cyberchondria**					.915		.916		.796
	CC^e^ 1	.904	3.421						
	CC 2	.912	3.329						
	CC 3	.898	3.127						
	CC 4	.854	2.202						
**Positive metacognition**					.898		.884		.710
	PM^f^ 1	.847	2.528						
	PM 2	.854	2.408						
	PM 3	.797	1.955						
	PM 4	.869	2.744						
	PM 5	.844	2.373						
**Health self-efficacy**					.870		.903		.718
	HS^g^ 1	.873	2.145						
	HS 2	.823	2.053						
	HS 3	.851	2.377						
	HS 4	.843	2.100						
**Subjective norm**					.912		.936		.850
	SN^h^ 1	.944	3.727						
	SN 2	.882	2.521						
	SN 3	.940	3.908						
**Health information similarity**					.880		.927		.731
	IS^i^ 1	.728	2.246						
	IS 2	.877	2.815						
	IS 3	.850	2.239						
	IS 4	.950	4.355						

^a^VIF: variance inflation factor.

^b^CR: composite reliability.

^c^AVE: average variance extracted.

^d^HIA: health information avoidance.

^e^CC: cyberchondria.

^f^PM: positive metacognition.

^g^HS: health self-efficacy.

^h^SN: subjective norm.

^i^IS: health information similarity.

Next, discriminant validity among the variables was verified, and the specific results are shown in Table 2. The square root of the AVE for all variables (on the diagonal) was greater than the Pearson correlation coefficients between the variables, indicating that the scales had satisfactory discriminant validity. A common method bias test was also conducted using the single-factor test. The variance explained by the first unrotated factor was 31.999%, which is below the critical threshold of 40%, indicating that common method bias is not an issue in this study [72].

**Table 2:** Discriminant validity analysis of constructs in study 1.

Constructs	HIA^a^	CC^b^	HS^c^	PM^d^	SN^e^	IS^f^
HIA	.860^g^	—^h^	—	—	—	—
CC	.410	.892^g^	—	—	—	—
HS	.346	.382	.848^g^	—	—	—
PM	.212	.343	.184	.843^g^	—	—
SN	.158	.137	.019	.651	.922^g^	—
IS	.339	.435	.291	.371	.246	.855^g^

^a^HIA: health information avoidance.

^b^CC: cyberchondria.

^c^HS: Health self-efficacy.

^d^PM: Positive metacognition.

^e^SN: subjective norm.

^f^IS: health information similarity.

^g^square roots of the average variance extracted.

^h^Not applicable.

#### Model Testing

The PLS algorithm was used to calculate the *R*² values for each variable, all of which were greater than the standard acceptance value of 0.1, indicating good predictive accuracy of the model [73]. Blindfolding was then conducted, and the results showed that the Stone-Geisser Q² values for all variables were greater than 0, indicating that the model effectively predicts the relationships between variables [74]. In addition, the SRMR value was 0.054, which is below the standard threshold of 0.08, the NFI value was 0.931, and the RMS θ value was 0.103. These results demonstrate that the model has good fit [75].

### Study 2

#### Participants and Procedure

The required sample size for the intervention study was estimated using GPower, with an effect size greater than 0.8 considered appropriate for the experimental study. Setting α=.05, 1-β=.8, and effect size=0.4, the minimum required sample size was calculated to be 44 [76].

The mindfulness training required participants to invest a significant amount of time, making it challenging to recruit a sufficient number of participants. To address this difficulty, our research team collaborated with students from 3 courses at 3 universities, encouraging them to invite their older family members (older than 60 years old and capable of independently using social media) to participate in the mindfulness training program. To motivate the students, the researchers offered course credits and a reward of approximately US $20. To improve the quality of the mindfulness training and the responses to the survey, the researchers informed participants before the experiment began that their responses would be kept strictly confidential and used only for academic purposes. Participants were also informed of the importance of honest responses and were made aware of their right to withdraw from the study at any time without conditions. Informed consent was obtained from all participants. The research was conducted in accordance with the standards of the Declaration of Helsinki.

We initially recruited 101 participants and divided them into 2 groups: group 1 (mindfulness training group, n=51) and group 2 (control group, n=50). The research process was conducted in 3 stages.

##### Stage 1 (t1)

At the start of the study, a survey measured participants’ levels of cyberchondria, health information avoidance, state mindfulness (Brown and Ryan [77]), and demographic variables (gender, age, income, and education). An independent samples *t* test showed no significant differences between the 2 groups for cyberchondria (M_group1_=2.848, M_group2_=3.110, *P*=.12, *t*_99_=–1.557), health information avoidance (M_group1_=3.063, M_group2_=3.384, *P*=.11, *t*_99_=–1.631), or state mindfulness (M_group1_=3.502, M_group2_=3.524, *P*=.77, *t*_99_=–.297).

##### Stage 2

Mindfulness training started 1 week after the initial survey to avoid bias from survey content. Group 1 underwent a short-term intervention based on the mindfulness-based stress reduction (MBSR) program (Kabat-Zinn [78]), while group 2 received no intervention. The training included five 10-minute audio sessions focusing on breathing, bodily sensations, thoughts, and emotions. Participants were instructed to complete a daily session at noon with randomly assigned recordings and record their practice (refer to Multimedia Appendix 1 for audio excerpts).

##### Stage 3 (t2)

Following 4 weeks of training, consistent with previous findings on mindfulness duration (Rooks et al [79]), 5 participants from group 1 and 2 from group 2 withdrew or failed to complete the study, leaving a final valid sample of 46 in group 1 and 48 in group 2. Cyberchondria, health information avoidance, and state mindfulness levels were reassessed. Reanalysis of initial data showed no significant differences between retained participants, ensuring data integrity.

### Ethical Considerations

This study strictly adhered to the ethical principles outlined in the Declaration of Helsinki and was approved by the Ethics Committee of the School of Journalism and Communication at Minjiang University (Ref: MJUCER20240107). Both substudies were conducted in strict accordance with relevant ethical guidelines to protect participants’ rights and ensure data confidentiality.

All participants provided informed consent before participating in the study. They were fully informed about the study’s objectives, procedures, data usage policies, and their right to withdraw from the study at any time without penalty. As the data were collected through questionnaires, the consent forms were obtained electronically.

To protect privacy and data security, all participant data were anonymized and deidentified during both the data collection and analysis processes. No personally identifiable information was recorded during the study, and all data were securely stored in an encrypted system to prevent unauthorized access.

Participants received appropriate compensation based on their involvement in the study. In substudy 1, a cross-sectional survey, participants were rewarded US $2 upon completing the questionnaire. In substudy 2, a 4-week longitudinal survey, participants received a gift valued at approximately US $20 after completing the study. These compensations were intended to acknowledge participants’ time and effort while avoiding undue influence on their decision to participate. This manuscript and its supplementary materials do not include any images or materials that could identify participants.

## Results

### Study 1

There were no missing values in the data. A bootstrapping test with a sample size of 5000 was conducted on the collected data to explore the path coefficients and their significance within the model. The final test results are presented in Tables 3 and 4.

The results of the data analysis indicate that cyberchondria exacerbates health information avoidance among older adults (β=.41, *P*<.001), supporting H1. From the perspective of internal factors, both positive metacognition and health self-efficacy have a positive influence on cyberchondria (β=.26, *P*=.002; β=.25, *P*<.001), which in turn further increases health information avoidance behavior (95% Boot CI 0.035-0.189; 95% Boot CI 0.043-0.185). Thus, H2a, H2b, H3a, and H3b are all supported. Regarding external environmental factors, the similarity of health information also significantly positively affects cyberchondria (β=.29, *P*<.001) and leads to health information avoidance through cyberchondria (95% Boot CI 0.063-0.191), supporting H4a and H4b. However, subjective norms did not show a significant direct relationship with cyberchondria (β=–.11, *P*=.13), and cyberchondria did not mediate the relationship between subjective norms and health information avoidance (95% Boot CI –0.102 to 0.016), leading to the rejection of H5a and H5b.

**Figure 2:**
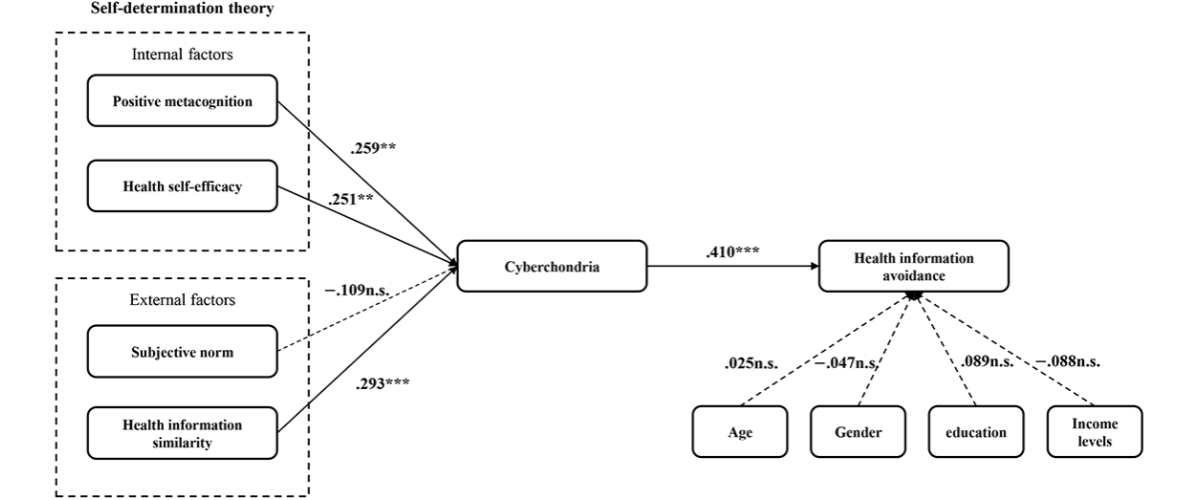
Result model of study 1: path analysis.**P<.01, ***P<.001, n.s. *P*>.05.

The results of study 1 indicate that cyberchondria significantly exacerbates health information avoidance among older adults, influenced by both internal factors (positive metacognition and health self-efficacy) and external factors (health information similarity). However, subjective norms did not have a significant effect on cyberchondria, suggesting that external social pressure is not a major driver of health information avoidance behavior in older adults. Based on these findings, the next study will focus on validating whether mindfulness training can effectively intervene in cyberchondria and the resulting health information avoidance behavior. The specific findings of study 1 are shown in Figure 2.

**Table 3:** Hypothesis testing results of study 1 (direct effect).

Direct effect	β values	*t* test (*df*)	*P* values
H1: CC^a^→HIA^b^	.410	7.016 (226)	<.001^c^
H2a: PM^d^→CC	.259	3.035 (226)	.002^e^
H3a: HS^f^→CC	.251	3.453 (227)	.001^e^
H4a: IS^g^→CC	.293	4.344 (227)	<.001^c^
H5a: SN^h^→CC	-.109	–1.507 (228)	.13

^a^CC: cyberchondria.

^b^HIA: health information avoidance.

^c^*P*<.001.

^d^PM: positive metacognition.

^e^*P*<.01.

^f^HS: health self-efficacy.

^g^IS: health information similarity.

^h^SN: subjective norm.

**Table 4:** Hypothesis testing results of study 1 (indirect effect).

Indirect effect	Effect value	*t* values (*df*)	95% CI	*P* values
H2b: PM^a^→CC^b^→HIA^c^	0.106	2.697 (220)	0.035 to 0.189	.007^d^
H3b: HS^e^→CC→HIA	0.103	2.821 (221)	0.043 to 0.185	.005^d^
H4b: IS^f^→CC→HIA	0.120	3.691 (221)	0.063 to 0.191	<.001^g^
H5b: SN^h^→CC→HIA	–0.045	1.459 (222)	–0.102 to 0.016	.15

^a^PM: positive metacognition.

^b^CC: cyberchondria.

^c^HIA: health information avoidance.

^d^*P*<.01.

^e^HS: health self-efficacy.

^f^IS: health information similarity.

^g^*P*<.001.

^h^SN: subjective norm.

### Study 2

Before hypothesis testing, the effectiveness of the mindfulness intervention was first examined. To control for the effects of covariates such as age, gender, and education level, an ANCOVA (analysis of covariance) was conducted to test the manipulation of state mindfulness. The analysis showed that the state mindfulness score of the intervention group was significantly higher than that of the control group (M_group1_=4.122, M_group2_=3.606, *F*_1,93_=16.866, *P*<.001). Subsequently, the within-group differences in t1 and t2 for both the intervention and control groups were compared. The results showed that the state mindfulness level of the intervention group at t2 was significantly higher than at t1 (M_t2_=4.122, M_t1_=3.502, *F*_1,96_=85.024, *P*<.001), while there was no significant difference between t1 and t2 in the control group (M_t2_=3.524, M_t1_=3.583, *F*_1,97_=.222, *P*=.64). In summary, the manipulation of state mindfulness was successful.

Next, ANCOVA was conducted to test H6a, with age, gender, education level, and income level as covariates. The results showed that there was no significant difference in the level of cyberchondria between t1 and t2 in the intervention group (M_t1_=2.848, M_t2_=2.685, *F*_1,96_=1.835, *P*=.18), indicating that H6a was not supported, meaning that mindfulness training did not improve the level of cyberchondria. Subsequently, H6b was tested using Hayes’ suggested Bootstrap method with SPSS (IBM Corp) process model 1 to examine the interaction effect, with 5000 bootstrap samples and a 95% CI. The data analysis showed that the interaction effect of mindfulness level and cyberchondria on health information avoidance was significant (effect=‒0.357, *P*=.002, 95% CI ‒0.580 to ‒0.131). Thus, H6b was supported.

**Figure 3:**
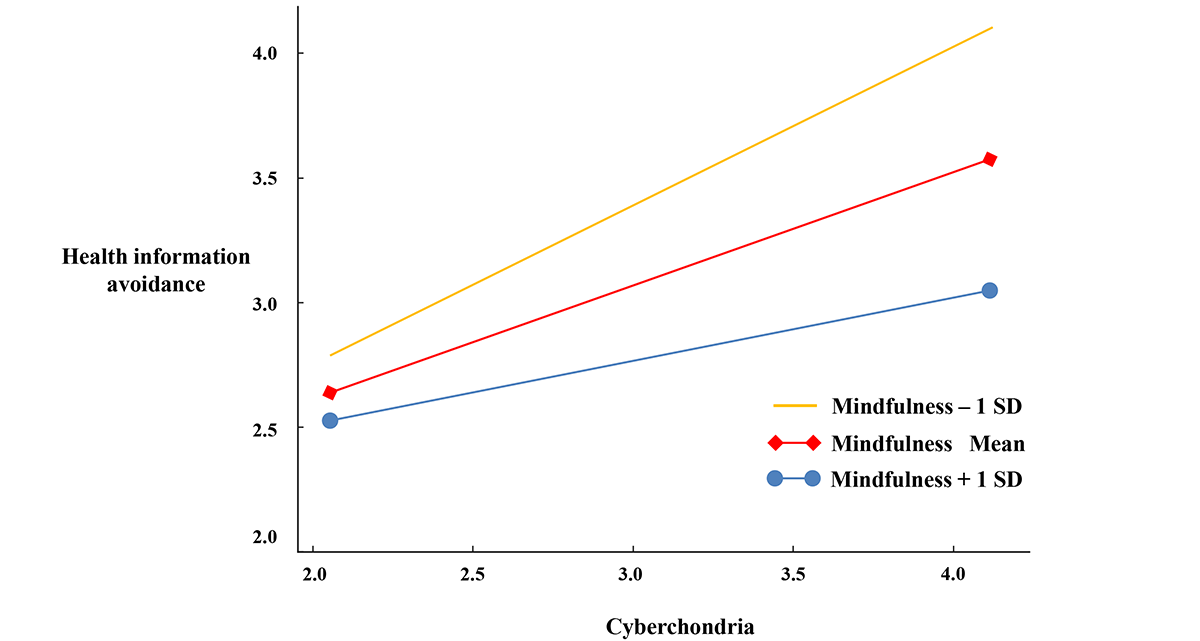
The Interaction effect of mindfulness and cyberchondria on health information avoidance.

In conclusion, the results of study 2 supported H6b but rejected H6a. Although mindfulness training did not directly reduce the level of cyberchondria, it effectively inhibited the transformation of cyberchondria into health information avoidance behavior (Figure 3).

## Discussion

### Principal Findings

Cyberchondria and health information avoidance have become increasingly pressing public health issues in the digital age, posing significant threats to the health of older adults. Therefore, exploring their underlying mechanisms and potential intervention strategies is of great theoretical and practical significance. Based on the self-determination theory, this study conducted 2 substudies to explore the causes of cyberchondria and health information avoidance among older adults, as well as the intervention effects of mindfulness training.

### Internal and External Factors Leading to Health Information Avoidance: Cyberchondria as the Mediator

The results reveal that internal factors (positive metacognition and health self-efficacy) and external factors (health information similarity) significantly predict cyberchondria among older adults, leading to health information avoidance.Positive metacognition fosters overthinking and health-related anxiety, as excessive focus on health concerns amplifies uncertainty. Previous research links positive metacognition to problematic behaviors [80], and this study confirms its role in exacerbating cyberchondria and health information avoidance. Health self-efficacy also influences cyberchondria. Although high self-efficacy promotes confidence in managing health, ambiguous health information undermines this confidence, driving excessive searches and heightening anxiety. This aligns with self-efficacy theory, where unresolved issues lead to repetitive checking and stress [81].

Health information similarity indirectly contributes to avoidance by increasing anxiety and cognitive overload. Repeated exposure to similar information, often driven by algorithms, makes it difficult to differentiate between diseases, heightening uncertainty and driving avoidance behaviors through cyberchondria [82]. However, subjective norms did not significantly affect cyberchondria, suggesting that older adults rely more on personal judgment and experience rather than external social influences [83,84]. This finding underscores the need for interventions targeting individual-level factors rather than external pressures.

### Mindfulness Training as an Effective Intervention for Cyberchondria and Health Information Avoidance

The results also indicate that while mindfulness training did not directly reduce the level of cyberchondria, it significantly mitigated the transition from cyberchondria to health information avoidance behavior. Mindfulness meditation, by enhancing emotional regulation and cognitive flexibility, helps older adults avoid being dominated by anxiety when faced with health information, even though it may not directly prevent the onset of cyberchondria. This effectively reduces their tendency to avoid health information. These findings are consistent with previous studies on the effects of mindfulness, supporting the conclusion that mindfulness training can help individuals better accept current emotions and reduce fear of future uncertainties [85]. Furthermore, our findings emphasize the role of mindfulness training in regulating health information behavior, demonstrating that it not only improves the emotional state of older adults but also increases their acceptance of health information, thereby reducing avoidance behaviors. This highlights the potential of mindfulness training to effectively break the vicious cycle of “anxiety–information search–greater anxiety–information avoidance.” Mindfulness can enable older adults to maintain a more balanced perspective when encountering health-related information, which prevents anxiety from escalating into avoidant behavior.

### Implications and Future Directions

#### This Study Makes Three Theoretical Contributions

First, it makes significant contributions to SDT by deepening the understanding of how internal and external factors influence health behaviors through psychological mechanisms. It validates the mediating role of cyberchondria in health information avoidance among older adults and demonstrates how positive metacognition, health self-efficacy, and health information similarity interact within this process. This fills a critical gap in previous research by uncovering the mediating mechanisms of health information avoidance and provides new insights into the cognitive-emotional processes of older adults, an information-vulnerable population. Furthermore, this study extends the applicability of SDT by highlighting the dynamic balance between intrinsic motivation and external support in older adults navigating complex digital health environments. In addition, this study systematically explores the role of mindfulness interventions in managing health information behaviors. While mindfulness did not directly reduce cyberchondria, it significantly inhibited its progression to information avoidance. By enhancing older adults’ capacity to cope with uncertainty and anxiety, mindfulness fosters a more open attitude toward health information. This expands mindfulness beyond emotional regulation to include its impact on complex health decision-making, providing theoretical support for its application in public health interventions. Finally, this study challenges the universal applicability of subjective norms in shaping health behaviors, as proposed in the theory of planned behavior [86]. It finds that older adults rely more on autonomous judgment than societal expectations due to their life experience and independence, emphasizing the importance of considering demographic differences and group heterogeneity in health behavior research.

#### Practically, This Study Offers Three Recommendations

First, for internal factors (eg, positive metacognition and excessive health self-efficacy), psychological education and cognitive behavioral therapy can help older adults reduce overfocus on health risks and avoid unnecessary health information searches [87]. Second, optimizing recommendation algorithms on health information platforms to reduce repetitive content and providing personalized information tailored to older adults’ health conditions can alleviate anxiety caused by information overload and similarity. Finally, enhancing digital health literacy through health seminars and digital skills training, combined with mindfulness programs, can offer a comprehensive intervention strategy to address health information avoidance. These measures collectively aim to reduce health anxiety, improve engagement with health information, and enable older adults to achieve proactive health management.

### Limitations

This study provides valuable insights into the causes of cyberchondria and health information avoidance among older adults and evaluates mindfulness meditation as an intervention. However, several limitations should be noted. First, the 4-week mindfulness intervention, while effective in the short term, does not address the sustainability of its effects. Future research should explore longer interventions and conduct follow-ups to assess lasting impacts. Combining mindfulness with approaches such as health education or social support may yield stronger and more enduring outcomes. In addition, while positive metacognition and health self-efficacy emerged as critical internal factors, specific intervention strategies for addressing these were not explored. Future research should investigate techniques such as cognitive behavioral therapy and health coaching to mitigate their negative impacts and further reduce health information avoidance. Expanding on these areas will help refine interventions and improve health management strategies for older adults.

### Conclusion

This study used 2 substudies to explore the causes of cyberchondria and health information avoidance among older adults, as well as the intervention effects of mindfulness meditation. Study 1 constructed an influence model based on self-determination theory, using a cross-sectional survey to verify that internal factors such as positive metacognition (β=.26, *P*=.002) and health self-efficacy (β=.25, *P*<.001), as well as external factors such as health information similarity (β=.29, *P*<.001), were significant predictors of cyberchondria among older adults, which in turn led to health information avoidance behavior (β=.41, *P*<.001). However, subjective norms did not show a significant association with cyberchondria among older adults (β=–.11, *P*=.13). Study 2 conducted a 4-week mindfulness intervention, demonstrating the effectiveness of mindfulness in inhibiting the transition from cyberchondria to health information avoidance (effect=–0.357, *P*=.002, 95% CI –0.580 to –0.131]). However, it was also found that mindfulness did not directly reduce the occurrence of cyberchondria (*P*=.18).

## Appendix

Multimedia Appendix 1 A: Demographic information B: Measures C: Study 2 Stimulus materials D: Checklist for Reporting Results of Internet E-Surveys (CHERRIES).

## Abbreviations

ANCOVA: analysis of covariance
AVE: average variance extracted
CR: composite reliability
MBSR: mindfulness-based stress reduction
PLS: partial least squares
SDT: self-determination theory
VIF: variance inflation factor

## Data Availability

The dataset of this study is accessible through science data bank website. In compliance with ethical guidelines and to protect participant privacy, all personally identifiable information has been removed from the dataset. Researchers are welcome to access the data through the provided link. For additional inquiries or specific questions about the dataset, please contact the corresponding author.
