# Supplementary material for: Mindfulness Intervention for Health Information Avoidance in Older Adults: Mixed Methods Study
Source: JMIR Public Health Surveill. 2025 Jan 28;11:e69554. doi: 10.2196/69554 (PMC11815307; doi:10.2196/69554)
Supplement: Multimedia Appendix 1 [file publichealth_v11i1e69554_app1.docx]

**Appendix A**

Table 1. Demographic information.

| Statistical items | Specific content | Statistical value | Percentage |
| --- | --- | --- | --- |
| Gender | Male | 91 | 38.56% |
|  | Female | 145 | 61.44% |
| Age | 60 ~ 65 | 178 | 75.43% |
|  | 66 ~ 70 | 41 | 17.37% |
|  | 71 ~ 75 | 13 | 5.51% |
|  | Over 76 | 4 | 1.69% |
| Educational background | Primary school | 66 | 28.00% |
|  | Middle school | 67 | 28.40% |
|  | High school | 64 | 27.10% |
|  | Undergraduate | 35 | 14.80% |
|  | Postgraduate | 4 | 1.70% |
| Social media usage duration/ day | Less than 1 hour | 20 | 8.48% |
|  | 1-3 hours | 92 | 38.98% |
|  | 3-5 hours | 67 | 28.39% |
|  | 5-7 hours | 45 | 19.07% |
|  | 7-9 hours | 8 | 3.39% |
|  | Over 9 hours | 4 | 1.69% |
| Income level/ month | Less than 1000 | 11 | 4.70% |
|  | 1001 ~ 3000 | 40 | 16.90% |
|  | 3001 ~ 5000 | 85 | 36.00% |
|  | 5001 ~ 7000 | 46 | 19.50% |
|  | 7001 ~ 9000 | 30 | 12.70% |
|  | More than 9001 | 24 | 10.20% |

**Appendix B**

**Measures**

**1. Health information avoidance**

Please indicate your agreement with the following statement. (1 = strongly disagree, 7 = strongly agree)

I would rather not know health information available online.

I avoid seeking health information online.

I believe that sometimes it's better not to know online health information.

I can recall situations where I have avoided health information.

I do not want to know health information from the internet.

**2. Cyberchondria**

Reflecting on times when you felt unwell and searched for information online, to what extent do the following statements describe your experience? (1 = strongly disagree, 7 = strongly agree)

I would prefer not to know health information available online.

I avoid accessing health information online.

I believe that sometimes ignorance is bliss when it comes to health information online.

I can recall instances where I avoided health information.

**3. Positive metacognition**

Please indicate your agreement with the following statement. (1 = strongly disagree, 7 = strongly agree)

Believing that my body is extremely healthy might be counterproductive.

Preparing for the worst regarding my health makes me feel safer.

I do not tend to think positively about my physical condition.

Frequently worrying about my health helps me cope with health issues.

Being overly optimistic about my health might lead to illness.

**4.** **Health self-efficacy**

Please indicate your agreement with the following statement. (1 = strongly disagree, 7 = strongly agree)

I have the ability to protect my health.

I have sufficient health knowledge.

I am capable of handling health issues well.

I can effectively protect my health.

**5.** **Subjective norm**

Imagine you are ill. To what extent do you agree with the following statements? (1 = strongly disagree, 7 = strongly agree)

My friends approve of me searching online for information related to my symptoms.

My family approves of me searching online for information related to my symptoms.

Other important people in my life approve of me searching online for information related to my symptoms.

**6.** **Health information similarity**

Please indicate your agreement with the following statement. (1 = strongly disagree, 7 = strongly agree)

I feel that the information provided by different health websites about a particular health issue is very similar.

When searching for health information, I find it difficult to identify differences between content from different sources.

The health information pushed online is often quite similar, making it hard for me to find anything new.

The descriptions of disease symptoms from different health information sources are all quite similar.

**7. State mindfulness**

Please indicate your agreement with the following statement. (1 = strongly disagree, 7 = strongly agree)

I find it difficult to stay focused on what's happening in the present.
I tend to walk quickly to get where I'm going without paying attention to what I experience along the way.
I rush through activities without being really attentive to them.
I find myself preoccupied with the future or the past.
I do jobs or tasks automatically, without being aware of what I'm doing.

**Appendix C**

Excerpt of the audio material.

"Now, gently notice that you are breathing. Feel the movement of your breath, feel the breath entering your body and then leaving your body. Do not think about whether your breathing is right or wrong, but directly feel your breath in any way that feels comfortable to you. If possible, notice the beginning, middle, and end of each inhale. Notice the interval between inhale and exhale. For the next few breaths, feel the beginning, middle, and end of each breath cycle. Now, prepare to expand your awareness to your thoughts. Notice how your thoughts come and go. Do not get caught up in what you are thinking about; when a thought arises, see it as just a thought, something fleeting. Sit quietly as your thoughts come and go, mindfully experiencing your thoughts. If you follow continuous thoughts too far, gently bring your attention back, notice your thoughts coming and going. Like leaves floating on a river or bubbles rising and disappearing in the air, observe your thoughts. Now, shift your attention to your emotions and allow them to come into your body and mind. You may feel upset, anxious, calm, sad, joyful, or fearful. At this moment, notice where the emotions originate in your body. Explore your emotions, and notice how we cling to pleasant emotions and strive to eliminate unpleasant ones. Whenever emotions or sensations make you uncomfortable, remember that you can always shift your focus to your breath, a safe space. Accept everything you feel. Feel everything at this moment, naturally."

**Appendix D**

**Checklist for Reporting Results of Internet E-Surveys (CHERRIES)**

| **Item Category** | **Checklist Item** | **Explanation** |
| --- | --- | --- |
| **Design** | Describe survey design | The survey was designed to investigate health information behaviors among older adults, with a specific focus on health information avoidance, cyberchondria, and mindfulness intervention outcomes. The target population consisted of individuals aged 60 years and older who were capable of independently completing an online survey. This demographic was chosen because of its unique challenges in navigating online health information and the potential psychological impact of such behaviors. The sample frame for the survey included older adults recruited through online platforms such as WeChat Moments, Douyin, and Jinri Toutiao  In Study 1, the participants were primarily sourced from online health-related communities and senior-focused networks, while Study 2 incorporated additional recruitment efforts through students from three universities who invited their older family members to participate in the mindfulness training program. All participants needed to meet the inclusion criteria, which included age over 60 and basic digital literacy to ensure they could access and complete the web-based questionnaire.  The survey used a convenience sampling approach, as participants were recruited from accessible online platforms and networks. This method allowed for efficient recruitment of a geographically dispersed population, although it may limit the generalizability of the findings. Efforts were made to diversify the sample by using multiple recruitment channels to include participants from various backgrounds. |
| **IRB (Institutional Review Board) approval and informed consent process** | IRB Approval | This study strictly adhered to the ethical principles outlined in the Declaration of Helsinki. The research protocol, covering both sub-study 1 and sub-study 2, was reviewed and approved by the Ethics Committee of the School of Journalism and Communication at Minjiang University (Approval Number: MJUCER20240107). Both studies were conducted in compliance with the institution's ethical research guidelines, ensuring that all measures were in place to protect the rights, safety, and well-being of participants. |
|  | Informed Consent | Participants provided informed consent electronically prior to their involvement in the research. They were thoroughly informed that the purpose of the study was to explore health information behaviors among older adults, with a specific focus on cyberchondria and health information avoidance. Participants were made aware that their participation in Sub-study 1 would require approximately 15 minutes, while Sub-study 2, involving mindfulness training, would span four weeks. Confidentiality and data protection were emphasized during the consent process, with assurances that all data would be anonymized, securely stored, and used solely for academic purposes. No personally identifiable information was collected or retained. Participants were also explicitly informed that their involvement was entirely voluntary and that they could withdraw from the study at any point without any consequences or penalties. These measures were designed to ensure transparency, respect for participant autonomy, and compliance with ethical standards throughout the research process. |
|  | Data Protection | To ensure participant privacy, all data collected in this study were anonymized and de-identified during both the collection and analysis stages. Data were stored securely on an encrypted server to prevent unauthorized access, and identifiable information was removed prior to analysis to guarantee anonymity. Access to the raw data was restricted exclusively to the primary researchers, further ensuring that participants’ personal information was fully protected throughout the research process. |
| **Development and pre-testing** | Development and testing | The survey used in this study was carefully developed based on existing validated scales and frameworks to ensure its reliability and relevance to the research objectives. Items were adapted from prior studies on cyberchondria, health information avoidance, and mindfulness to fit the context of older adults. To ensure clarity and appropriateness, the questionnaire underwent a pilot testing phase before its formal implementation. A small group of older adults (N = 10) who met the inclusion criteria participated in this pilot study. Feedback from these participants was used to refine the wording of questions, optimize the flow of the survey, and ensure that the language was clear and accessible for the target population.  Additionally, the technical functionality and usability of the electronic questionnaire were tested during the pilot phase. This included ensuring compatibility across different devices (e.g., smartphones, tablets, and computers) and verifying that the survey could be completed without technical difficulties. The pilot test confirmed that the questionnaire interface was user-friendly and the platform supported seamless navigation for participants, minimizing any potential technical barriers. These steps ensured that the survey was well-suited for the older adult population and capable of collecting high-quality data. |
| **Recruitment process and description of the sample having access to the questionnaire** | Open survey versus closed survey | Open survey |
|  | Contact mode | The initial contact with potential participants was made entirely through the Internet. The recruitment messages contained detailed information about the purpose of the study, eligibility criteria, and instructions for accessing and completing the web-based questionnaire. Participants were provided with a link to the secure survey platform, where they could independently complete the questionnaire online. This approach ensured broad accessibility and convenience, particularly for older adults who frequently use digital platforms for communication and information-seeking. |
|  | Advertising the survey | The survey was announced and advertised exclusively online through health-related forums, social media platforms (such as WeChat Moments, Douyin, and Jinri Toutiao). The recruitment messages were tailored to the target audience, emphasizing the study’s focus on older adults' health information behaviors and mindfulness. The announcements highlighted the eligibility criteria, participation benefits, and the estimated time required to complete the survey, encouraging voluntary participation. |
| **Survey administration** | Web/E-mail & Context | The e-survey for Study 1 was distributed through the Wenjuanxing platform (an online survey tool commonly used in China). Participants accessed the survey via a direct link to the platform, where their responses were automatically recorded and stored in a secure database.  For Study 2, the survey link hosted on the Wenjuanxing platform was shared with participants through WeChat. Participants accessed the questionnaire by clicking the link, and their responses were similarly captured and stored automatically in the Wenjuanxing system, ensuring efficiency and data accuracy. |
|  | Mandatory/voluntary | Voluntary survey |
|  | Incentives | In study 1, participants were rewarded $2 upon completing the questionnaire.  In study 2, participants received a gift valued at approximately $20 after completing the study. |
|  | Time/Date | Study 1: 18/01/2024 to 16/02/2024  Study 2: 20/03/2024 to 17/04/2024 |
|  | Randomization of items or questionnaires | To minimize potential biases in responses, the survey included features to randomize or alternate the order of certain items. |
|  | Number of Items | Study 1: 30 items  Study 2: 19 items |
|  | Completeness check | To ensure the completeness and consistency of responses, the survey utilized built-in functionality provided by the Wenjuanxing platform. Mandatory fields were implemented for all essential questions, requiring participants to provide a response before proceeding to the next section of the survey. If a participant attempted to skip a required item, a prompt highlighted the unanswered question and directed them back to complete it. Additionally, non-response options such as "Not applicable" or "Prefer not to answer" were provided for sensitive or context-specific questions, ensuring participants could submit a valid response without discomfort. The completeness check was conducted dynamically during the survey process, minimizing missing data and improving the quality of the collected responses. |
|  | Review step | The survey included a review step to allow respondents to review and modify their answers before final submission. Participants could use the "Back" button to navigate to previous sections of the questionnaire and adjust their responses if necessary. Additionally, at the end of the survey, a summary page displayed all their answers, providing participants with the opportunity to confirm or correct their entries before submitting. |
| **Response rates** | Unique site visitor | To ensure the accurate tracking of unique site visitors and participation rates, the survey employed both IP address monitoring and cookie-based identification through the Wenjuanxing platform. Each participant's IP address was logged to prevent duplicate submissions from the same device or network. |
|  | Completion rate (Ratio of users who finished the survey/users who agreed to participate) | Study 1: 71.95%  Study 2: 92.157% |
| **Preventing multiple entries from the same individual** | IP check | The survey utilized IP address monitoring to identify and prevent potential duplicate entries from the same user. Through the Wenjuanxing platform, multiple entries originating from the same IP address were restricted within a 24-hour period. This ensured that users could not access and complete the survey multiple times within a short timeframe. If duplicate entries with the same IP address were detected over a longer period, only the first completed entry was retained for analysis, while subsequent entries were excluded. |
|  | Registration | The survey was conducted as an open survey, allowing participants to access it through a general link distributed via social media platforms (e.g., WeChat) and email invitations. Although the survey was publicly accessible, measures were implemented to prevent duplicate entries and maintain data quality. |
| **Analysis** | Handling of incomplete questionnaires | Only completed questionnaires were included in the final analysis to ensure data quality and reliability. Participants who did not complete all sections of the questionnaire or terminated the survey early were excluded from the dataset. |
|  | Questionnaires submitted with an atypical timestamp | To ensure the validity of the collected data, questionnaires submitted with atypical timestamps, indicating unusually short completion times, were excluded from the analysis. |
|  | Statistical correction | No statistical correction methods, such as weighting of items or propensity scores, were applied in this study. As the sample was recruited using convenience sampling through online platforms, we acknowledge that the results may not be fully representative of the broader older adult population. However, efforts were made to mitigate potential biases by recruiting participants from diverse sources, including online communities, social media platforms, and offline networks, to ensure a certain level of variability in the sample. The findings should be interpreted with caution, considering the limitations of the sampling method. |
